# Nanobubbles of Oxygen, Air, and Ozone Gas for the Degradation of Reactive and Cationic Dyes from Wastewater

**DOI:** 10.1021/acs.langmuir.5c02324

**Published:** 2025-08-06

**Authors:** Athanasios T. Varoutoglou, Konstantinos N. Maroulas, Margaritis Kostoglou, Evangelos P. Favvas, Dimitra A. Lambropoulou, Athanasios C. Mitropoulos, George Z. Kyzas

**Affiliations:** † Hephaestus Laboratory, School of Chemistry, Faculty of Sciences, 609468 Democritus University of Thrace, GR-65404 Kavala, Greece; ‡ Laboratory of Chemical Technology, School of Chemistry, 37782 Aristotle University of Thessaloniki, Thessaloniki 541 24, Greece; § Institute of Nanoscience and Nanotechnology, NCSR “Demokritos”, TermaPatriarchou, Grigoriou and Neapoleos, Aghia Paraskevi 153 41, Attica, Greece; ∥ Laboratory of Environmental Pollution Control, School of Chemistry, Aristotle University of Thessaloniki, Thessaloniki 541 24, Greece; ⊥ Center for Interdisciplinary Research and Innovation (CIRI-AUTH), Balkan Center, Thessaloniki 57001, Greece

## Abstract

The presence of dyes in industrial effluents causes significant environmental harm. Traditional wastewater treatment technologies are insufficient to remove dyes rapidly. This study examined the degradation efficiency of dyes (Methylene Blue (MB) and Remazol Brilliant Blue R (RBBR)) by using nanobubbles (NBs) of ozone (O_3_), oxygen (O_2_), and air. For their generation, hydrodynamic cavitation was selected. The impacts of the flow rate, pH, reaction kinetics, and initial pollutant concentration were investigated. As expected, the flow rate affected NB size and concentration, impacting pollutant removal efficiency. ζ potential showed that O_3_@NBs achieved the highest absolute value of 27.8 mV at pH 7.5, exhibiting the best stability and performance. Experimental results show that the implantation of O_3_@NBs rapidly removes 100% of MB and RBBR within 15 min, independent of pollutant concentration or pH. O_2_ and air NBs had lower removal efficiencies, indicating the higher oxidative potential of O_3_@NBs. In addition, the soluble O_3_@NBs managed to degrade 40 and 65% of the total organic content for MB and RBBR, respectively. Kinetics analysis showed that all NBs follow a first-order kinetic model. The stability of produced NBs was explored over the span of 1 year, revealing O_2_@NBs as the most stable. Exploring the application in real textile wastewater showed that O_3_@NBs can effectively be employed to obtain clear water, since it removed >70% of both the dye and total dissolved solids present in the solution. Also, scavenger studies revealed that hydroxyl radicals are highly responsible for the degradation of both MB and RBBR. Overall, this work provides a mechanistic understanding of the reactivity of O_3_@NBs, O_2_@NBs, and Air@NBs and sheds light on the importance of nanobubble features and reaction parameters in optimizing advanced oxidation processes for wastewater treatment applications.

## Graphical abstract

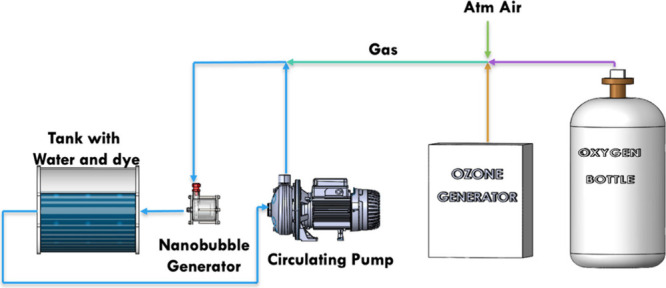

## 1 Introduction

The alarming surge in water contamination, a pressing issue that has escalated in recent years due to the far-reaching effects of global industrialization, demands immediate and concerted attention. This problem is not isolated to a single sector but is a collective result of various industries, such as textiles, agriculture, and transportation, all contributing to the release of toxins into the ecosystem. The urgency of this issue cannot be overstated, and immediate action is imperative.^1^ These contaminants might be organic or/and inorganic. Toxic heavy metals, such as mercury, lead, chromium, and arsenic, are the most common inorganic contaminants. Organic pollutants primarily include organic matter, nutrients, colors, antibiotics, herbicides, pesticides, POP (persistent organic pollutants), etc.^2^ Among these pollutants, textile dye molecules significantly contribute to the occurrence of hazardous and carcinogenic chemicals in freshwater bodies.^3^ A significant proportion (about 60–70%) of synthetic organic dyes are used to color various materials, from artificial and natural fibers to plastics, leather, paper, mineral oils, waxes, and, occasionally, foods, drug, cosmetic items, and fuel-marking industries. This widespread use underscores the pervasiveness of the problem.^4^ These dyes have a low affinity for substrates, meaning they do not bind strongly to the materials to which they are applied, resulting in 5 to 1500 mg/L concentrations. Dye-containing effluent can contaminate adjacent water bodies and pose a significant hazard to human health and aquatic life owing to dyes’ poisonous and carcinogenic properties.

In addition to carcinogenic risks, colors can cause allergic reactions, resulting in skin irritation and dermatitis in sensitive individuals. Certain dyes’ toxicological profile is further influenced by their heavy metal concentration, which includes lead, chromium, and cadmium.^5^ Also, these wastewaters often contain a mixture of cationic and anionic dyes.^6^ The simultaneous presence of the latter results from the diverse range of dyeing processes and fiber types used in manufacturing. Their coexistence poses significant challenges for wastewater treatment. Methylene Blue (MB) is a dark green solid that can irritate the respiratory tract, skin, and eyes and induce cyanosis and blue skin discoloration.^7^ It is widely used in cotton dyeing and wood industries. Exposure to this material may cause irreparable eye damage in people and animals as well as burns, nausea and vomiting, increased sweating, and psychological disorders. Remazol Brilliant Blue R (RBBR) is an anionic dye resistant to chemical oxidation due to its aromatic anthraquinone structure via resonance, negatively impacting humans and the environment. These potential health risks underscore the gravity of the situation and the need for immediate action.^8^ However, tons of wastewater are dumped into the environment every year from various industrial sources.

To reduce the environmental impact of dyeing wastewater, industries typically use wastewater treatment processes such as biological treatment, adsorption, membrane filtration, ion exchange, coagulation precipitation, and advanced oxidation processes (AOPs) to remove or reduce the concentration of pollutants.^9,10^ However, these methods have several flaws, including high costs, no pollutant decomposition (particularly in adsorption methods, where disposal of the used adsorbent remains a problem), the need for special conditions to work correctly (particularly in biological processes), and the possibility of the formation of toxic byproducts. For instance, biological treatment methods require specific environmental conditions and a long retention time, making them less efficient for large-scale industrial applications. Additionally, in membrane technology, one of the leading separation technologies, dye aggregation and salting-out, can contribute to membrane fouling, ultimately rendering the membrane element ineffective.^11,12^ Adsorption on the other hand merely transfers contaminants onto a surface, requiring further treatment.^13^ These limitations underscore the need for better, more effective solutions.^14^ Therefore, further research is imperative to identify efficient, cost-effective, and operationally feasible solutions.

One of the most novel and promising ways to degrade dyes from wastewater is the utilization of nanobubbles (NBs).^15^ There are several types of micro- and nanobubble generators, such as gas–water circulation, pressurization–decompression, spiral flow, ejector-type, and cavitation-based approaches.^16–18^ Hydrodynamic cavitation, which is often implemented in the form of a venturi, stands out for its efficiency in large-scale wastewater treatment due to its compact design, low energy demand, and capacity to produce high-density, fine bubbles via pressure-driven cavitation.^19–21^ Unlike acoustic cavitation, which is expensive and difficult to scale, the venturi-type provides a realistic and scalable approach for producing effective NBs for versatile applications.^22^ The method involves the creation of a high-pressure gradient in a fluid, often through the passage of the fluid through narrow channels or specially designed nozzles. When the fluid experiences a swift transition from its high-pressure to low-pressure state, dissolved gases are nucleated to form cavitation bubbles.^23,24^ This method facilitates the controlled, homogeneous generation of bubbles and at the same time produces bubbles of exceptional stability.

Also, NBs exhibit excellent endurance and Brownian motion, which allow them to cover a wider surface area, whether rising to the surface or staying in the water.^25^ In addition, they feature a large interfacial area and a slow bubble rise pace. Furthermore, the collapse of nanobubbles creates reactive oxygen species (ROS), which include singlet oxygen, superoxide, and hydroxyl radicals, as well as shock waves that can contribute to the degradation of pollutants.^26^ Similarly, the high mass transfer rate and abundance of free radicals in nanobubbles are widely utilized for the removal of heavy metal ions and the degradation of organic pollutants in industrial wastewater.^27^ So far, NBs have been used alone or paired with other methods for the degradation of pharmaceuticals,^14,28^ bisphenols,^28^ and dyes,^29^ as well as for the oxidation of heavy metals such as As­(III) to As­(V).^30^ There are various ways to generate NBs. Compared to others, hydrodynamic cavitation (HC) technology offers the following advantages: low cost, easy maintenance, high efficiency, ease of industrial production, simplicity of operation, ability to enhance chemical reactions, and absence of secondary pollutants.^31^ Venturi tubes are the most used HC devices, because they give optimal cavitation intensity. Furthermore, they are simple to use and have low maintenance expenses.^32^ The only potential limitation for scaling up is the amount of pump energy required to generate the pressure differentials required for nanobubble formation, to achieve small NBs size (<200 nm).^33^

NBs have also shown promise in different applications because of their unique properties. They are effective in cleaning wastewaters by breaking down pesticides, mycotoxins, and heavy metals through the production of reactive oxygen species.^34^ They also help seeds germinate, plants grow, and crops become better by delivering more nutrients and stimulating beneficial changes in soil microenvironments. Recently, their usage has expanded in the synthetic route of multiple materials. For example, it was shown that incorporating NBs in graphene oxide synthesis not only increased significantly the surface area (∼2.5 times) but also induced microporosity to the final material and increased oxygen content.^35^

The current study examines the degradation of MB by employing air, O_2_, or O_3_ gases to produce NBs, since O_2_ has a mild oxidizing character and O_3_ has a higher oxidation capacity. It aims to understand the influence of various parameters, including gas flow rate, pH, initial concentration, and kinetics, on the degradation process as well as on the properties of the produced NBs. In addition, their stability over a span of 1 year is tested, and their potential application with real wastewater, along with the degradation mechanism. This is the first time such a comprehensive approach was followed to link the physicochemical properties of NBs with the degradation efficiency, and it will provide valuable insights into the potential of NBs for wastewater treatment in real-life scenarios.

## 2 Experimental section

### 2.1 Materials

Methylene Blue dye was purchased from PanReac (Chicago, IL, USA). Sodium hydroxide solution (0.1 M), hydrochloric acid solution (0.1 M), and 2-propanol were purchased from Merck (Darmstadt, Germany). The O_2_ and O_3_ gases used were of high purity (99.99%). Remazol Brilliant Blue R dye was purchased from Acros Organics (Geel, Belgium). The industrial effluents from the dyeing reactor were kindly supplied by the local textile dying industry (Thessaloniki, Greece).

### 2.2 NBs Generator

The NBs used in this research were produced by a generator whose three-dimensional representation with the necessary equipment is shown in Figure 1. The nanobubbles are made through a hydrodynamic cavitation phenomenon at a pressure of ∼3 bar. Hydrodynamic cavitation can be described by Bernoulli’s equation (eq 1):^36^

1 $$P+\frac{1}{2}{\rho U}^{2}=C\left(\mathrm{constant}\right)\to U^{2}+\frac{2P}{\rho }=\frac{2C}{\rho }$$

where *P* is the pressure, *U* is the flow velocity of water at a point, and *p* is the density of the liquid. Therefore, the pressure takes negative values (negative pressure) in conditions where the water flow velocity $>\sqrt{\frac{2C}{\rho }}$ , and then the cavitation phenomenon occurs, where shear stresses combined with under-pressure conditions fragment the gas phase of the aqueous solution, creating nanoscale bubbles.

**Figure 1:**
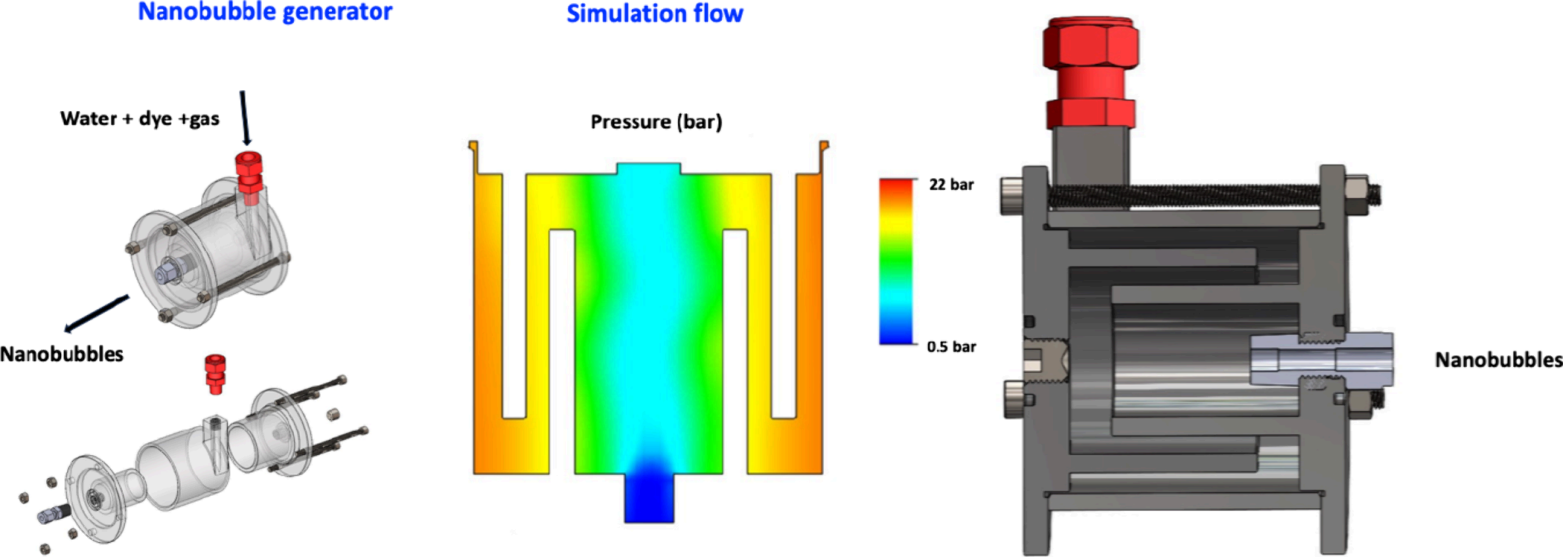
NB generator.

### 2.3 Instrumental Analysis

To determine the distribution of size and the concentration of NBs in the tap water, a nanoparticle detection analyzer was used (NanoSight LM-10, Malvern Instruments Ltd., Malvern, Worcestershire, U.K.). ζ potential values were measured using a Malvern Zetasizer Nano ZS, via dynamic light scattering. For nanoparticles in liquids, the Brownian motion rate was not affected by particle density and was related only to viscosity and temperature. On the other hand, NBs in liquid were illuminated by a laser, and the analyst used a video imaging device to record the Brownian motion of the NBs. The size of the nanoparticles was calculated according to the nanoparticle motion rate by using the Stokes–Einstein equation. The measurement range for the size distribution was from 20 to 1000 nm, and the measurement range for the number of MNBs was 10^6^–10^9^ bubbles/mL. The concentrations of MB and RBBR solutions were measured with a UV spectrophotometer (Hitachi U-2900) at λ_max_ 663 nm^37^ and at 590 nm,^8^ respectively. Total organic carbon (TOC) for aqueous solutions was determined with a TOC-L analyzer (Shimadzu Corp., Japan).

### 2.4 Experimental Design

Experiments were performed in the tank of the nanobubble generator with a total volume of 5 L. The pH of the solution was measured to be 7.5. Tests were carried out on five dye solutions of different concentrations (10, 20, 30, 40, and 50 ppm) and four different flow rates (0.3, 0.6, 0.9, and 1.2 L/min) at three pH values (4, 7.5, and 12). The kinetics of dye degradation was examined in the 0–50 min range.

## 3 Results and Discussion

### 3.1 NBs Properties

Many factors influence the efficiency of hydrodynamic cavitation, including the following: (i) the inlet pressure, (ii) the liquid’s physical and chemical properties, (iii) the cavitation tube construction, and (iv) the gas solubility. When a liquid flows at a high speed, HC occurs because the pressure at a given place goes below the vapor pressure instantly^38^ (see Figure 1). Figure 2 represents the size distribution of the three types of NBs used. According to Air@NBs results (Figure 2a), the flow rate seems to affect their size and concentration.^39^

**Figure 2:**
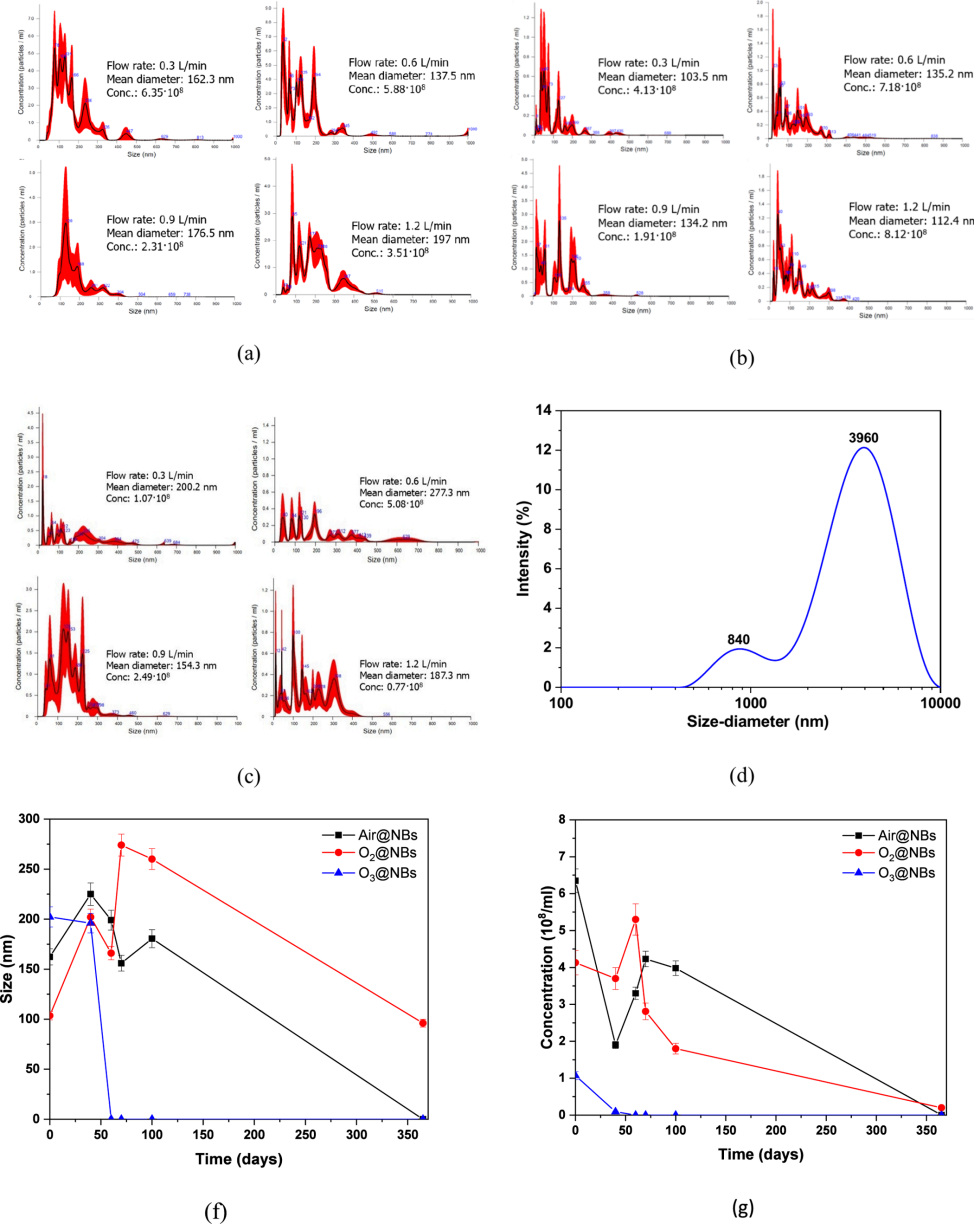
Size distributions and concentrations of NBs (a–c). DLS measurements (size, nm) of MNBs (d) (tap water, pH 7.5 at 20 °C). Effects of time regarding their (f) size and (g) concentration in tap water.

By increasing the flow rate, the number of NBs decreases. However, this trend does not apply to their mean diameter, with a flow rate of 0.6 L/min, resulting in the smallest diameter at 137.5 nm. In contrast, O_2_ NBs (O_2_@NBs) do not follow the same pattern (Figure 2b), where the lowest flow of 0.3 L/min provides the smallest size of 103.5 nm and the highest flow rate (1.2 L/min) the more considerable concentration of 8.12 × 10.^8,40^ Finally, it can be observed (Figure 2c) that the O_3_@NBs have again a completely different pattern, where the flow rate of 0.3 L/min generates the most NBs. Comparing the sizes of NBs, it can be concluded that their size and concentration are relative to the weight and the solubility of the gases.^41^ O_2_, being the lighter gas, provides the smallest and the least NBs, and that of O_3_ is precisely the opposite. In cases that involve hydrodynamic cavitation, it was observed that high levels of gas flows promote gas entrapment within the liquid, possibly due to oversaturation, enhancing the likelihood of bubble coalescence.^38^ The process creates larger bubbles through the aggregation of smaller bubbles. This is responsible for the reduced NBs concentration and a broader size distribution. For comparison purposes, micro-/nanobubbles using O_3_ gas (O_3_@MNBs) were generated using MNB generator with a flow rate of 0.3 L/min and their size was calculated using Malvern Instruments Series, Nano-ZS with multipurpose titrator. The presented DLS curve (Figure 2d) is created from the average value of three sets of runs, with each set being the average result of ten continuous measurements. The population/concentration of O_3_@MNBs was approximately 100 × 10^5^ NBs/mL, most of them being microbubbles that dominate the solution (3960 nm), whereas a second peak at 840 nm indicates the presence of NBs.

The stabilities of all three NBs were tested in a span of 1 year, and the results regarding their size and concentration are presented in Figure 2f,g, respectively. For Air@NBs during the first 40 days, an increase was shown in their size to ∼230 nm, followed by a decline from the 70th until the 365th day where they vanished. A similar trend was observed for the O_2_@NBs, which showed the biggest increase in the 100th day, reaching 275 nm, more than 2.5 times their initial size. Lastly, the O_3_ NBs were the least stable, remaining stable in the first 40 days, and after 20 days they disappeared. As far as their concentration, O_3_@NBs showed 0.09 × 10^8^ bubbles/mL during the first 40 days, a number 10 times lower than its initial concentration. Air@NBs and O_2_@NBs followed again the same trend, having an initial decrease of their concentration, which was then significantly increased at the 70th day. From this point onward, the decline started, with O_2_@NBs existing in the aqueous solution after a year, at a value of 0.2 × 10^8^ bubbles/mL. Initially, the increase in NB size and concurrent decrease in concentration over the first 40 days likely resulted from coalescence and Ostwald ripening, where smaller bubbles dissolve and redeposit onto larger ones, consistent with mechanisms proposed in both Eklund and Swenson and Soyluoglu et al.^42,43^ These results come in agreement with similar studies that examined the stability of NBs in a long period of time.^44,45^

ζ potential reflects the adsorption of ions in bulk solutions, leading to an electrical charge on the external bubble surface. It is also a critical parameter in the study of NBs, as it directly influences their stability and behavior. Based on the literature, it is caused by the combination of ion adsorption on the bubble surface and the formation of counterions on the interior surface.^46^ According to Figure 3a, the absolute value of ζ potential is higher for O_3_@NBs at 27.8 mV, then for O_2_@NBs, and last for Air@NBs. These values are in agreement with the literature^47–49^ and could be accounted for the fact that ozone is a highly reactive and very soluble gas, which produces hydroxyl radicals and O_2_. Also, higher ζ potential value reflects a larger electrostatic stability, less aggregation, and increased dispersibility in a solution.^50,51^ In Figure 3b, the ζ potential of the O_3_@NBs becomes increasingly negative as pH increases, reaching approximately −48.97 mV at pH 12. This trend is due to the increased dissociation of hydroxyl groups (OH^–^) at higher pH, which enhances negative charge accumulation on the bubble surface.^52^ At lower pH, fewer hydroxyl ions are present, leading to a less negative ζ potential and ultimately to a less stable colloidal system.^53^ Higher reactivity and dye molecule interaction can be attained with the O_3_@NBs, enhancing the oxidative degradation efficiency. In the presence of OH, ozone decomposition can react via the following mechanism:^54^

2 $$O_{3}+{\mathrm{OH}}^{-}\to O_{2}+{{\mathrm{HO}}_{2}}^{-}$$

3 HO2−+O3→O•2−+OH•+O2

4 O3+O•2−→O•3−+O2

5 $${O_{3}}^{-}+H_{2}O\to {\mathrm{HO}}^{•}+{\mathrm{OH}}^{-}+O_{2}$$

**Figure 3:**
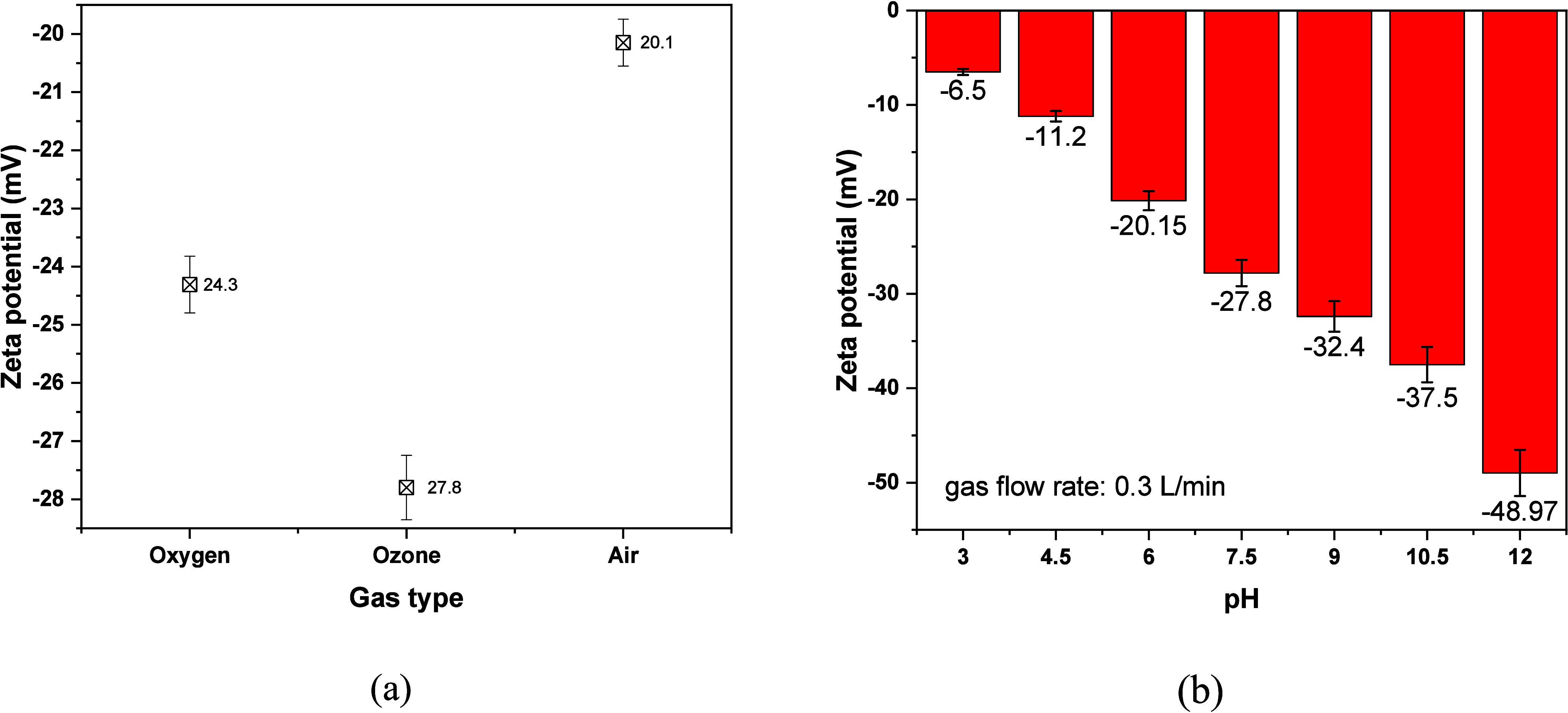
ζ potentials of (a) NBs using different gases at pH 7.5 and (b) O_3_@NBs at different pH values (tap water at 20 °C).

### 3.2 Effect of Flow Rate

Experiments were also run at different flow rates (0.3–1.2 g/L), keeping constant the initial concentration (10 mg/L), pH (7.5), and temperature (20 °C), and the results are presented in Figure 4. The optimal gas flow rate can increase the number of gas nuclei in the fluid and lower the cavitation threshold, producing more cavitation bubbles.^55^ Under specific conditions, the quantity of cavitation bubbles determines the severity of the reaction. However, excessive gas inlets will unavoidably alter the physical properties of the fluid and increase its compressibility.^56^ In addition, increasing the gas flow rate could possibly hinder NBs breakage and, therefore, the generation of hydroxyl radicals. NBs generate ^•^OH under high temperature and pressure during shrinking, and such radicals produce oxygen radicals. Bubble collapse and thermal decomposition of water vapor molecules cause ROS production.^57^ Figure 4a shows the effect of the flow rate of gases toward MB removal. O_3_@NBs can eliminate >99% of MB in all flow rates, possibly due to the increased hydroxyl radicals, compared to the other NBs where the efficiency was significantly lower. O_2_@NBs had the best flow rate at 0.3 L/min, eliminating ∼30%, while Air@NBs managed to remove ∼20% at 0.6 L/min. For these two specimens, the size of the NBs seems to impact their removal efficiency, since both had the lowest size at these flow rates, according to Figure 2a. Thus, for O_3_@NBs and O_2_@NBs 0.3 L/min was selected as the optimal flow rate and 0.6 L/min for Air NBs. For RBBR, the concentration of NBs is observed to be the driving force for the degradation of the pollutant. With the collapse of NBs, a huge number of hydroxyl radicals are generated and intense mechanical action is performed, increasing the breakdown efficiency of RBBR.^32^ For this reason, 0.3 L/min flow rate was selected for Air- and O_3_@NBs, while for O_2_ 1.2 L/flow rate was selected for the rest of the RBBR experiments.

**Figure 4:**
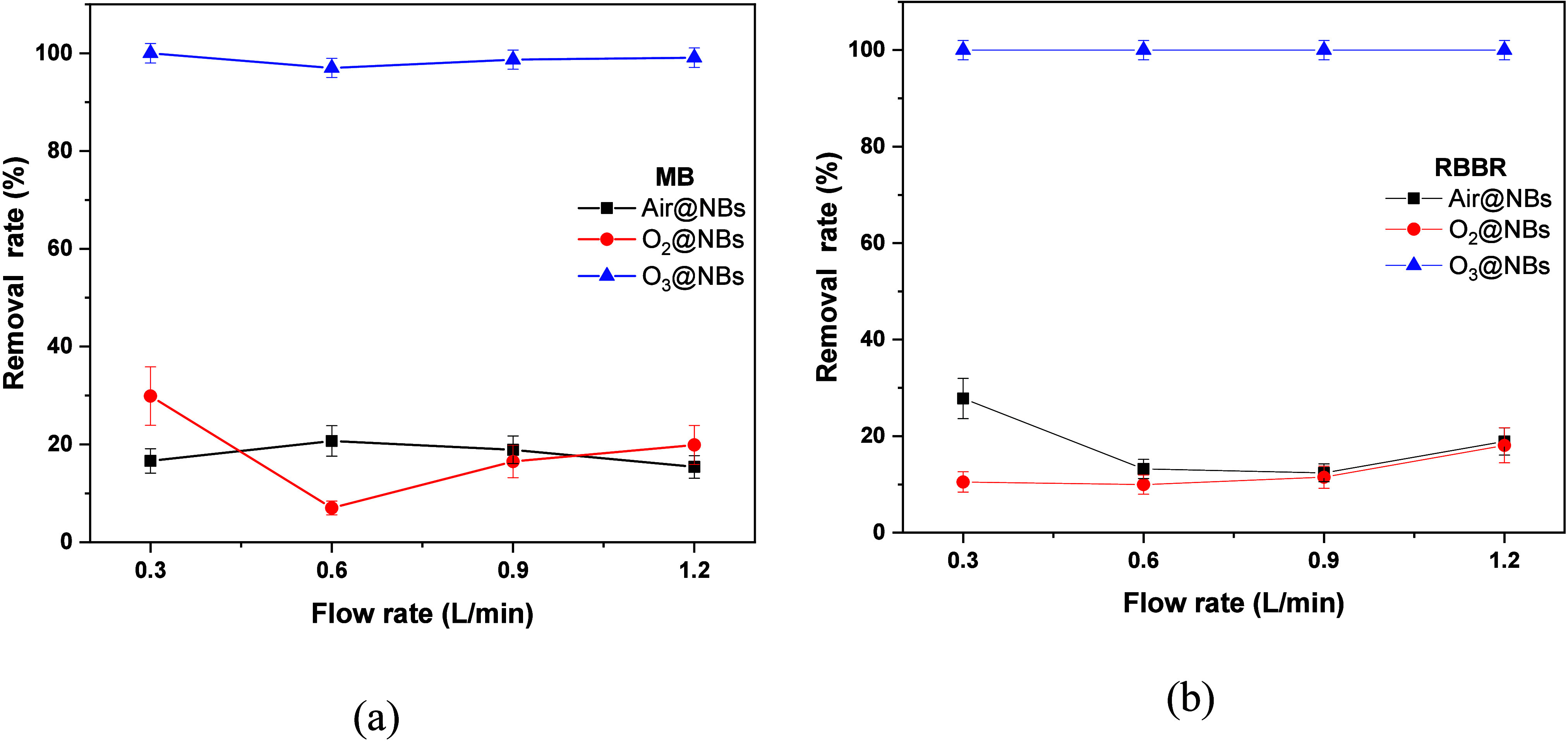
Effects of the flow rate in the degradation of (a) MB and (b) RBBR.

### 3.3 Effect of pH

The initial pH of the solution plays a critical role in the degradation mechanism of both MB and RBBR because it influences the generation of reactive radicals and the chemical activity of the nanobubbles generated throughout the process.^58^ For this series of experiments, the concentration of both dyes remained constant (10 mg/L) and the optimal flow rates obtained from the Section 3.2 were selected for each pollutant. In Figure 5a it can be observed that pH does not affect the degradation of MB for O_3_@NBs, where the removal (%) was kept at 100%. In contrast, Air- and O_2_@NBsper performed better at alkaline levels (pH 12). MB has a p*K*_a_ of 3.8,^59^ which means that in this pH range is negatively charged. It is reported that when NBs are generated in acidic media, they tend to have bigger sizes.^60^ As previously stated, for MB degradation the size of NBs had a significant role and it was observed that smaller NBs performed better and this could explain why pH 12 had better results than neutral and acidic media.^61^ For this reason, it was selected for the rest of the MB experiments. Regarding RBBR, pH 4 exhibited the best results for all three NBs and therefore was chosen for the RBBR experiments. O_3_@NBs kept their brilliant performance, not affected by changes of pH values, demonstrating 100% removal of the dye. So possibly NBs attach on both dyes’ surface through H-bonding between the water molecules surrounding them and the amino units of the dyes.

**Figure 5:**
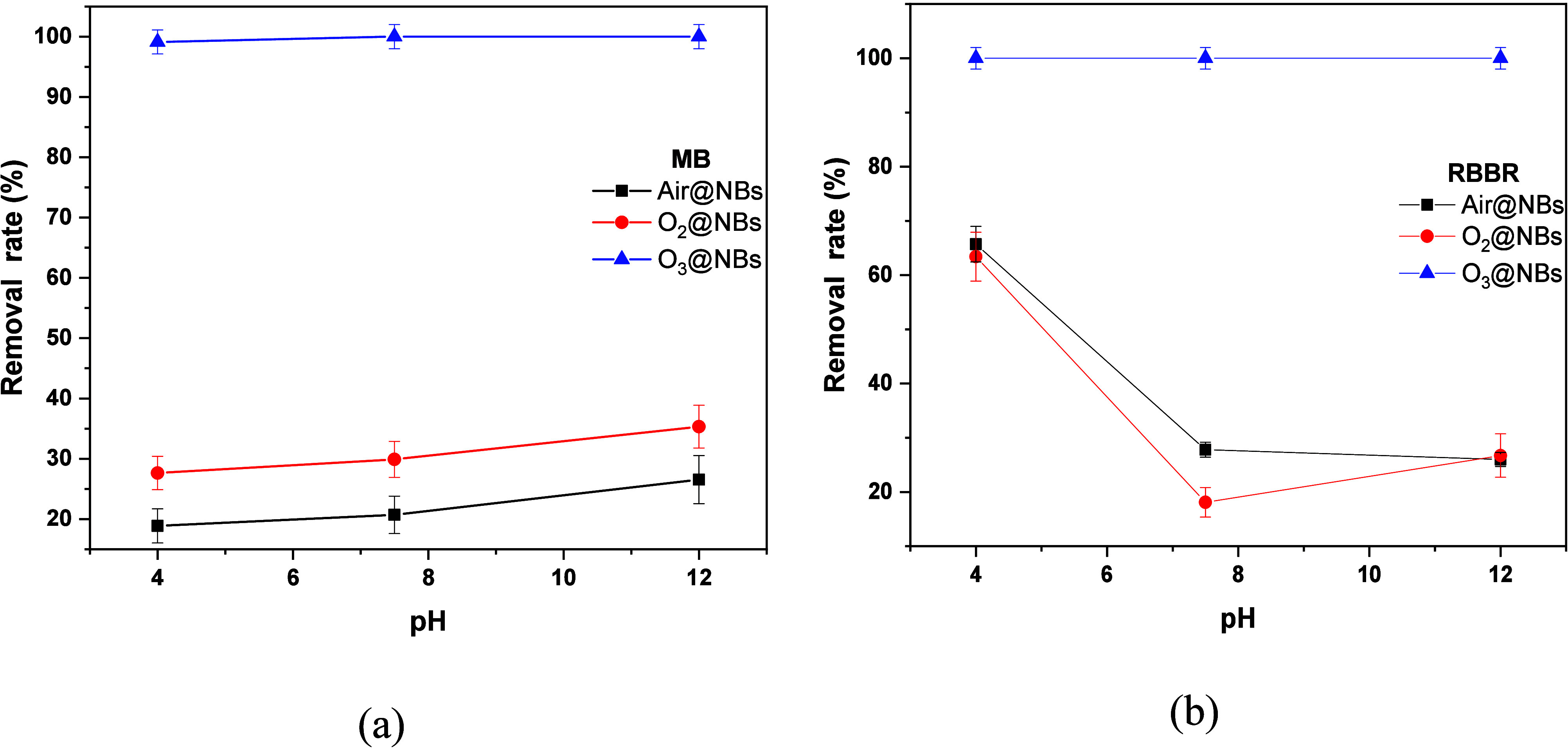
Effects of the pH in the degradation of (a) MB and (b) RBBR.

The enhanced degradation of RBBR in contrast to the behavior toward MB, for both Air@NBs and O_2_@NBs can be understood through the structural differences present in the two dyes. RBBR, being an anionic dye with sulfonic acid groups, at low pH values, will exist in its protonated form. This results in increased adsorption onto negatively charged NBs, thus making the degradation process easier.^61^ At high values of pH, its sulfonic groups will become deprotonated, leading to repulsive forces and reducing degradation efficiency. That observation clarifies the vastly increased degradation efficiency for RBBR at low values of pH, in contrast to a significant drop in such efficiencies at high values of pH.^62^ For MB, as previously stated, it remains negatively charged in the studied pH range, thus having electrostatic repulsion with the NBs. The latter justifies the low efficiency of O_2_ and Air@NBs.

### 3.4 Effect of Degradation Time

The kinetic degradation analysis of MB and RBBR is presented in Figure 6a,b, respectively. The initial concentration was kept at 10 mg/L for both dyes, while flow rate and pH values were selected from the previous experiments.

**Figure 6:**
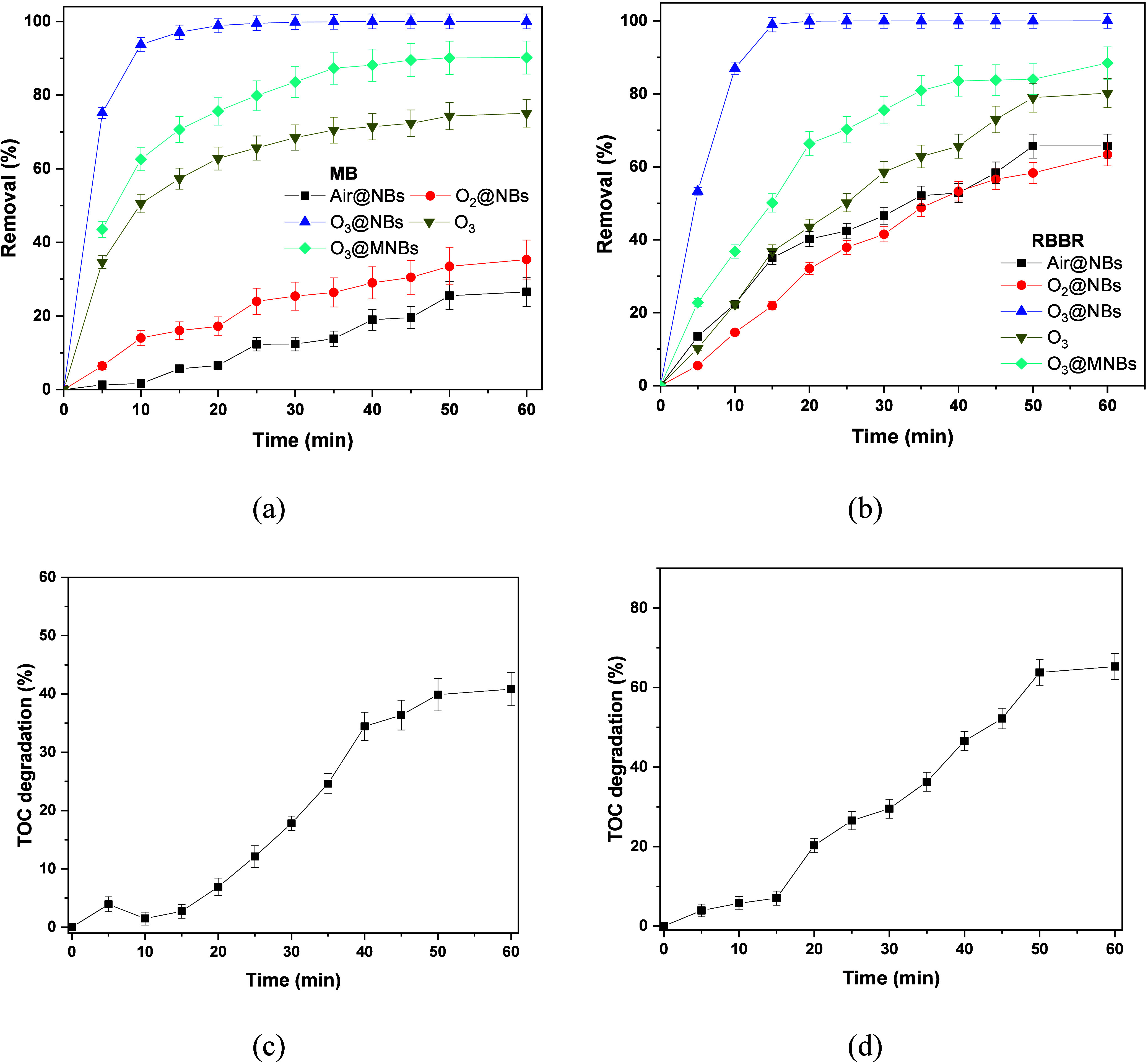
Kinetics on the degradation of (a) MB and (b) RBBR. Kinetics on the degradation of TOC for (c) MB and (d) RBBR, in the case of O_3_@NBs.

For MB, efficiency in decoloration with Air@NBs and O_2_@NBs is steady but not exceeding 40% even at 60 min, indicative of a poor oxidative effect. For both of these NBs, the plateau was reached within 50 min. O_3_@NBs showed a fast degradation within 10 min, eliminating over 90% of MB, which was entirely degraded at 20 min. For this reason, additional experiments using ozonation and aqueous O_3_@MNBs were performed to further evaluate the importance of NBs. As can be seen in Figure 6a, both ozonation and O_3_@MNBs perform better than Air- and O_2_@NBs eliminating ∼78 and 90%, respectively. Nevertheless, their equilibrium was met at around 45 min, which is 25 min longer than that of O_3_@NBs, underscoring the enhanced performance of the latter.

However, degradation of RBBR with Air@NBs and O_2_@NBs presented better results. This degradation is slow-paced, with over 60% decoloration in the same 60 min time frame. O_3_@NBs performed well again, but this time 100% degradation was achieved in the first 15 min. All of these observations confirm the high oxidation efficiency with O_3_@NBs. This is in line with other reported studies, which used the same concentration of MB.^63–65^ However, this performance was not better than that of the O_3_@NBs, which managed to remove 100% of the total dye and almost 95% from the first 10 min. In the case of RBBR, ozonation achieved a degradation of ∼80%, but again with a much slower rate than that of the rate of O_3_@NBs. O_3_@MNBs achieved a remarkable 86% removal with a rapid degradation efficiency in the first 20 min, which reached a plateau 25 min later. These results make the use of O_3_@NBs a more economic and viable option for decolorization of wastewaters.

To assess the mineralization efficiencies of MB and RBBR, the total organic carbon (TOC) degradation rates were measured for O_3_@NBs. Panels c and d of Figure 6 show that the removal efficiency of TOC rises with reaction time for both dyes. Therefore, the results show that MB and RBBR or other organic contaminants systems are successfully destroyed.^66,67^ These values are close to those reported by other studies that employed NBs for the degradation of organic pollutants.^68,69^

### 3.5 Effect of Initial Concentration

Panels a and c of Figure 7 present the effect of the initial dye concentration. As can be seen, O_3_@NBs eliminated 100% of both dyes in all concentrations studied, confirming the high oxidative potential. The decomposition of dyes possibly occurred through electrophile attack and ozone decomposition in water to produce hydroxyl radicals. On the other hand, Air@NBs and O_2_@NBs showed negligible oxidative activity for MB and RBBR. The results showed that O_2_@NBs could degrade 5–10 mg/L of both dyes’ solutions. Air@NBs had similar performance with O_2_@NBs for MB, while it showed better performance for RBBR, eliminating ∼15 mg/L.

**Figure 7:**
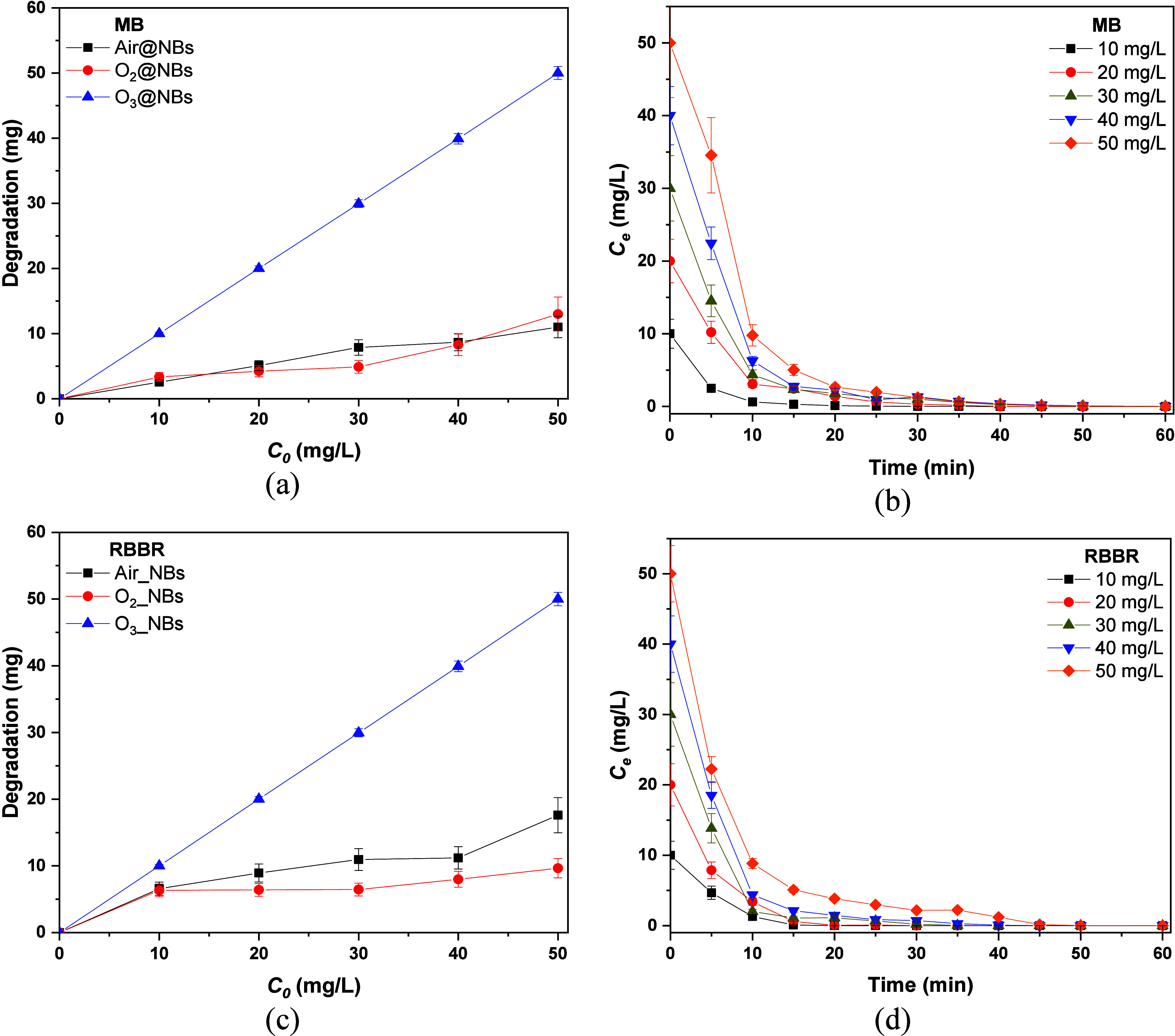
Effects of the initial concentration of dyes on the degradation of (a) MB and (c) RBBR and on the kinetics of O_3_@NBs toward (b) MB and (d) RBBR.

Since O_3_@NBs removed 100% in each case, additional kinetic experiments were carried out to gain a better understanding of their performance. In the case of MB in Figure 7b, the pollutant concentration rapidly decreased at 10 min with less than 10% present. A similar pattern was observed for RBBR in Figure 7d, where most of the dye degraded in the first 10 min. Thus, the initial concentration of MB and RBBR did not affect the degradation efficiency.^70,71^

### 3.6 Degradation Kinetic Data Analysis

The entire system, including the recirculation branch with pumps and other devices, can also be considered a closed system. This means that independently from the way of decomposition, it can be considered to behave as a batch reactor. The degradation reaction rate must be affected by the dye concentration and ozone concentration. It appears that the decomposition is induced by the nanobubbles and not by dissolved ozone. All of the present experiments (except those for air) have been made with the same recirculation rate, so the nanobubble concentration can be assumed as the same and its participation to reaction rate cannot be estimated. Therefore, the reaction rate, which depends solely on the dye concentration, will be evaluated in the present work. The analysis will be performed in terms of the fraction of the dye reacted *X* (=1 – *C*/*C*_0_, where *C* is the dye concentration and *C*_0_ its initial value). The remaining fraction 1 – *X* is the fraction of the dye that remained unreacted, and this is equal to *C*/*C*_0_ pointing its relevance to the actually measured quantity *C*. The above argument suggests that the fit by the models must be done in terms of 1 – *X*. Another advantage of this quantity is that a reaction of first order is independent from the initial concentration *C*_0_. Two tools will be used for the modeling of the reaction kinetics. The first is the first order reaction for which

6 $$\frac{dC}{dt}=-\mathrm{kC}\Rightarrow 1-X=\exp\left(-\mathrm{kt}\right)$$

The second tool is the reaction of arbitrary order *n*:^72^

7 $$\frac{dC}{dt}=-{\mathrm{kC}}^{n}\Rightarrow 1-X={\left(1-{\mathrm{kC}}_{0}^{n-1}\left(1-n\right)t\right)}^{1/\left(1-n\right)}$$

Let us start with the case of air and oxygen nanobubbles for MB. The extent of reaction is small (*X* less than 0.35). For such a small extent of reaction, every reaction mechanism can be approximated by a linear time dependence. This can be confirmed by the Taylor expansion of the exponential in eq 6 (i.e., 1 – *X* = 1 – *kt*). The first order reaction rate is assumed to hold here as the simplest one. The fitting of data with straight lines led to *k* = 0.0045 min^–1^ for air and *k* = 0.0071 min^–1^ for oxygen. The fitting curves are shown in Figure 8a. The extent of reaction is clearly larger in the case of RBBR, so the straight-line approximation does not hold. The fitting by the first order model appears in Figure 8b, and it led to *k* = 0.018 min^–1^ for air and *k* = 0.02 min^–1^ for oxygen.

**Figure 8:**
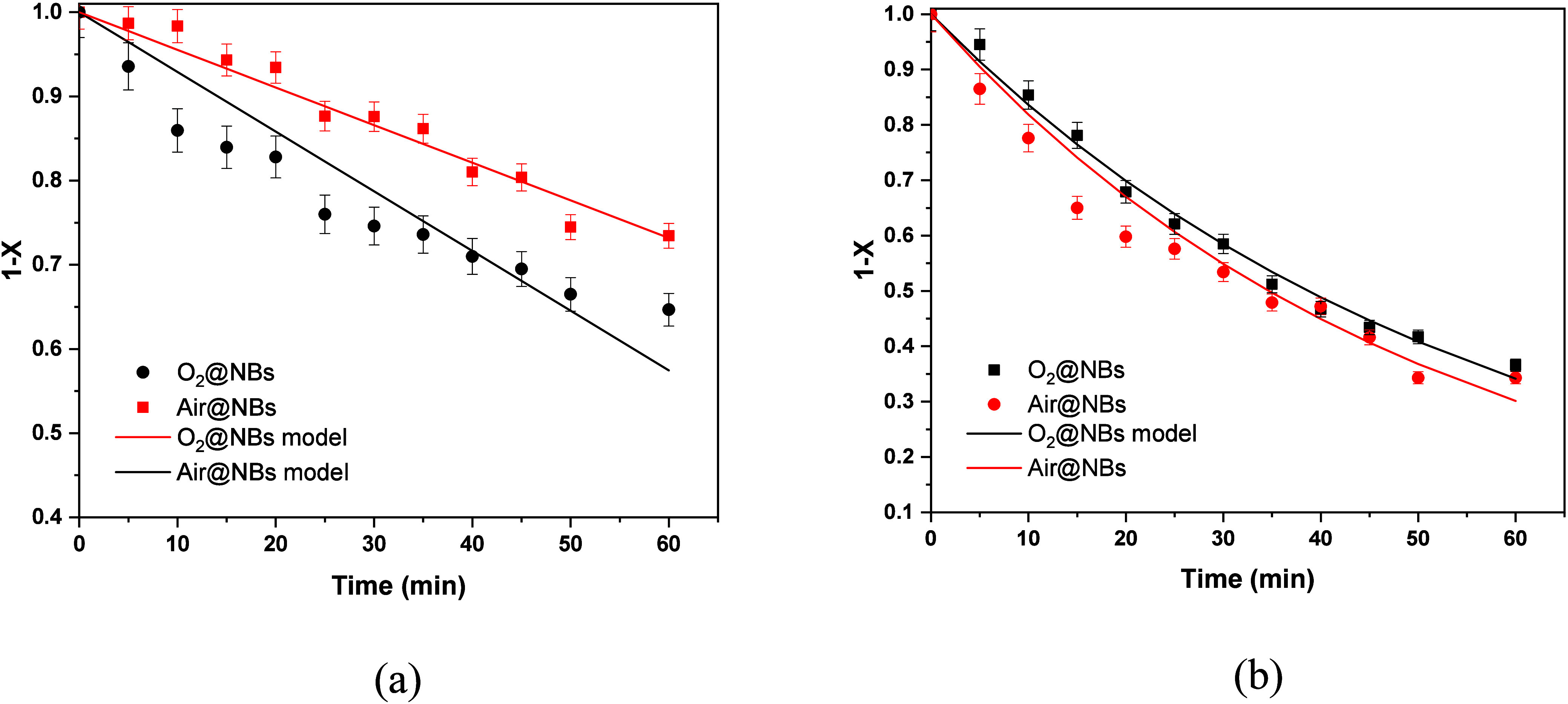
Evolution of the unreacted fraction for (a) MB and (b) RBBR using air or oxygen nanobubbles. Experiments and models.

Let us continue by analyzing the experiments with ozone nanobubbles. The curves 1 – *X*(*t*) for MB and concentrations *C*_0_ = 20, 30, and 40 mg/L are very close to each other. This is a strong indication of a first order reaction, taking into account the experimental uncertainties. So, the average value of 1 – *X* is calculated for these three initial concentrations, and it is fitted with the exponential of eq 6. The comparison between experiments and the model appears in Figure 9a, confirming the first order reaction rate for these three initial concentrations. The resulting reaction constant is *k* = 0.16 min^–1^. The cases with initial concentrations *C*_0_ = 10 ppm and *C*_0_ = 50 mg/L are modeled by using the arbitrary order reaction rate shown in eq 7. The comparison between the model and experiment appears in Figure 9b. The parameters found are *n* = 1.086, *kC*_0_^0.086^ = 0.3 min^–1^ for *C*_0_ = 10 mg/L and *n* = 0.77, *kC*_0_^–0.23^ = 0.123 min^–1^ for *C*_0_ = 50 mg/L. It is noticed that for *C*_0_ = 10 mg/L the exponent *n* is very close to unity.

**Figure 9:**
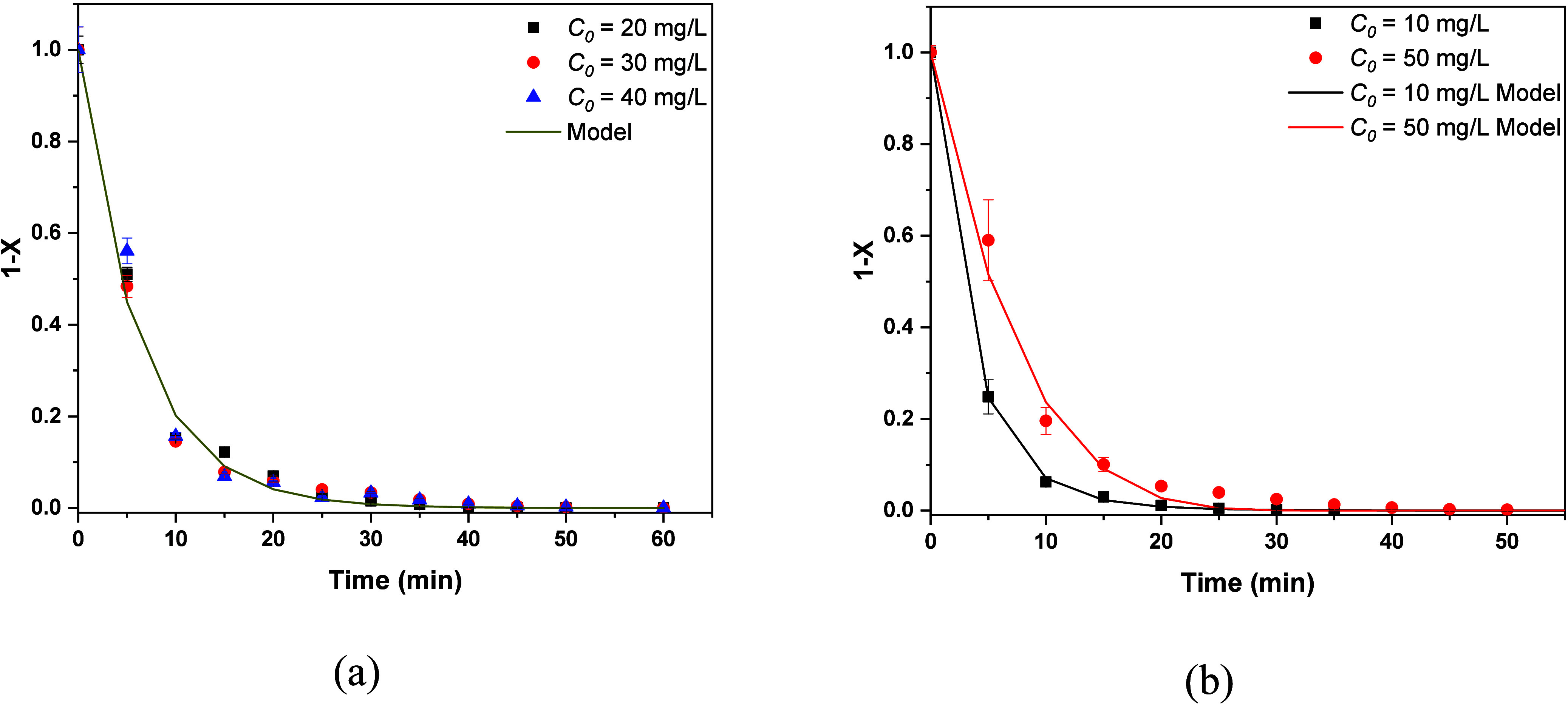
Evolution of the unreacted fraction for MB using ozone nanobubbles for (a) three initial concentrations and (b) two initial concentrations. Experiments and models.

Regarding the RBBR, it is observed that all the curves 1 – *X*(*t*) are close to each other except the one that corresponds to *C*_0_ = 50 mg/L. The four curves are simulated using first order reaction kinetics. It is noticed that, in this case, the kinetics constant is the same for all of the initial concentrations (*C*_0_). This is the confirmation that the first order model holds. The comparison between the model and data appears in Figure 10a. The resulting kinetic constant is *k* = 0.188 min ^–1^. The case with initial concentration *C*_0_ = 50 mg/L is fitted using the arbitrary order model, and the result is shown in Figure 10b. The constants found are *n* = 1.28 and *kC*_0_^0.28^ = 0.2 min^–1^. All of the fitting procedures have been performed based on the least-squares criterion.

**Figure 10:**
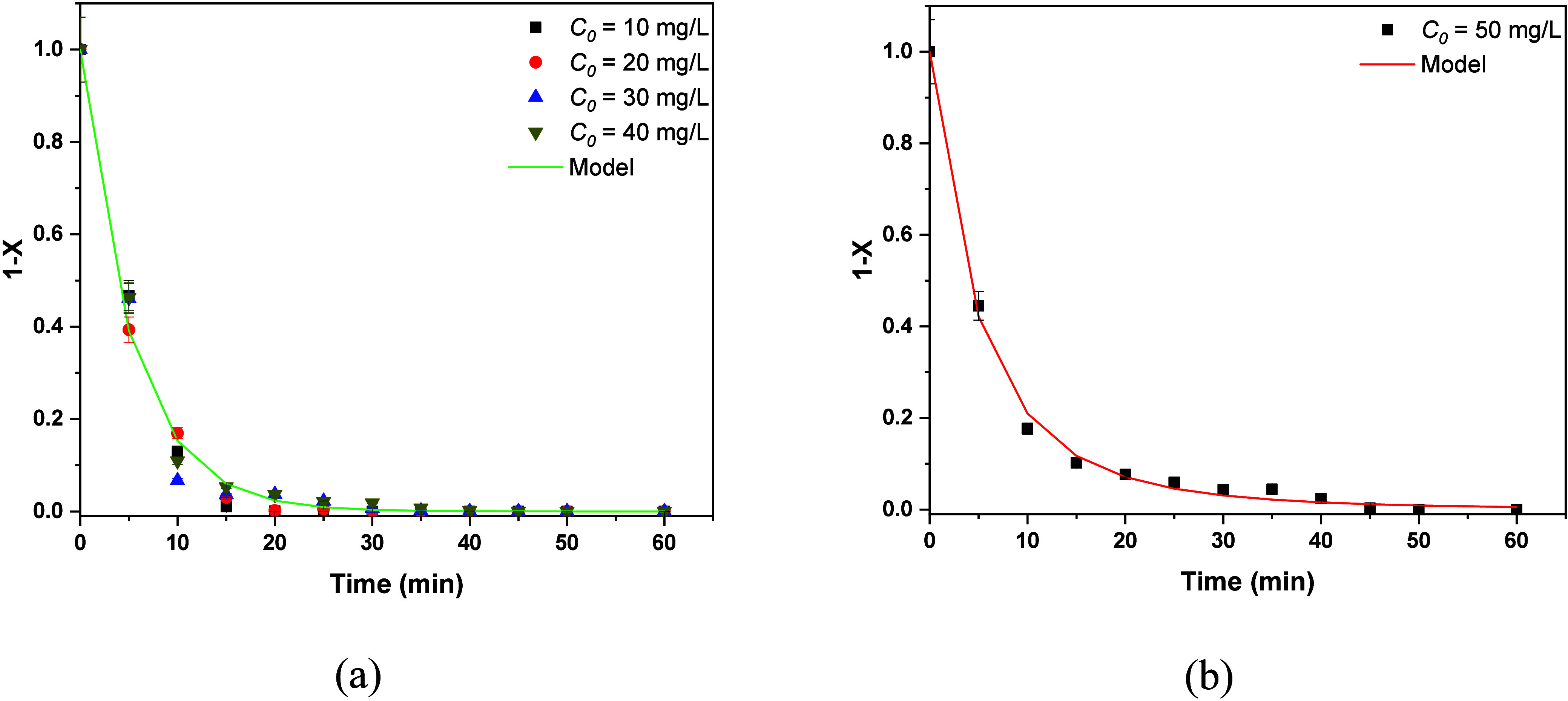
Evolution of unreacted fraction for RBBR using O_3_@NBs for (a) four initial concentrations and (b) 50 mg/L initial concentration. Experiments and models.

Summarizing, most of the data in the present work follow first order kinetics with respect to the dye. Notable exceptions are the large initial concentrations of 50 mg/L where much more complex phenomena than a single decomposition may occur. The resulting first order reaction constant is more than an order of magnitude larger for ozone than for oxygen and air. The decomposition kinetics is somewhat faster for RBBR compared to MB.

### 3.7 Degradation Mechanisms

The degradation mechanisms of dyes by O_3_-, O_2_-, and Air@NBs differ in their generation of ROS. Hydrodynamic cavitation enhances gas–liquid contact and stabilizes nanobubbles, prolonging ROS activity.^73^ O_3_@NBs degrade dyes via direct ozone decomposition in water and indirect pathways as shown in eqs 2–5, yielding abundant hydroxyl radicals for rapid, nonselective oxidation. O_2_ nanobubbles generate weaker ROS through electron transfer:^26,57^

8 O2+e−→O•2−

with limited ^•^OH formation via protonation:

9 O•2−+H+→HO•2

resulting in lower degradation efficiency than those of O_3_@NBs. Air@NBs (N_2_/O_2_) follow oxygen’s pathways but with reduced ROS yield, since N_2_ generates hydroxyl radicals only in acidic media.^74,75^

To further validate the aforementioned assumptions, radical quenching experiments were performed to examine the possible free radicals that were generated. Thus, 2-propanol (1 mM) was used as trapping agent for hydroxyl radical.^54,76^ In the case of MB (Figure 11a), without any scavenger, 100% removal efficiency was achieved within 45 min. However, in the presence of 2-propanol the degradation of the particular dye was hindered significantly, eliminating only 60% of MB after 1 h of the process. A similar behavior was observed for RBBR dye, where only 50% was degraded in the presence of 2-propanol. These results strongly indicate both the presence and the significance of ^•^OH in the degradation mechanism using O_3_@NBs.

**Figure 11:**
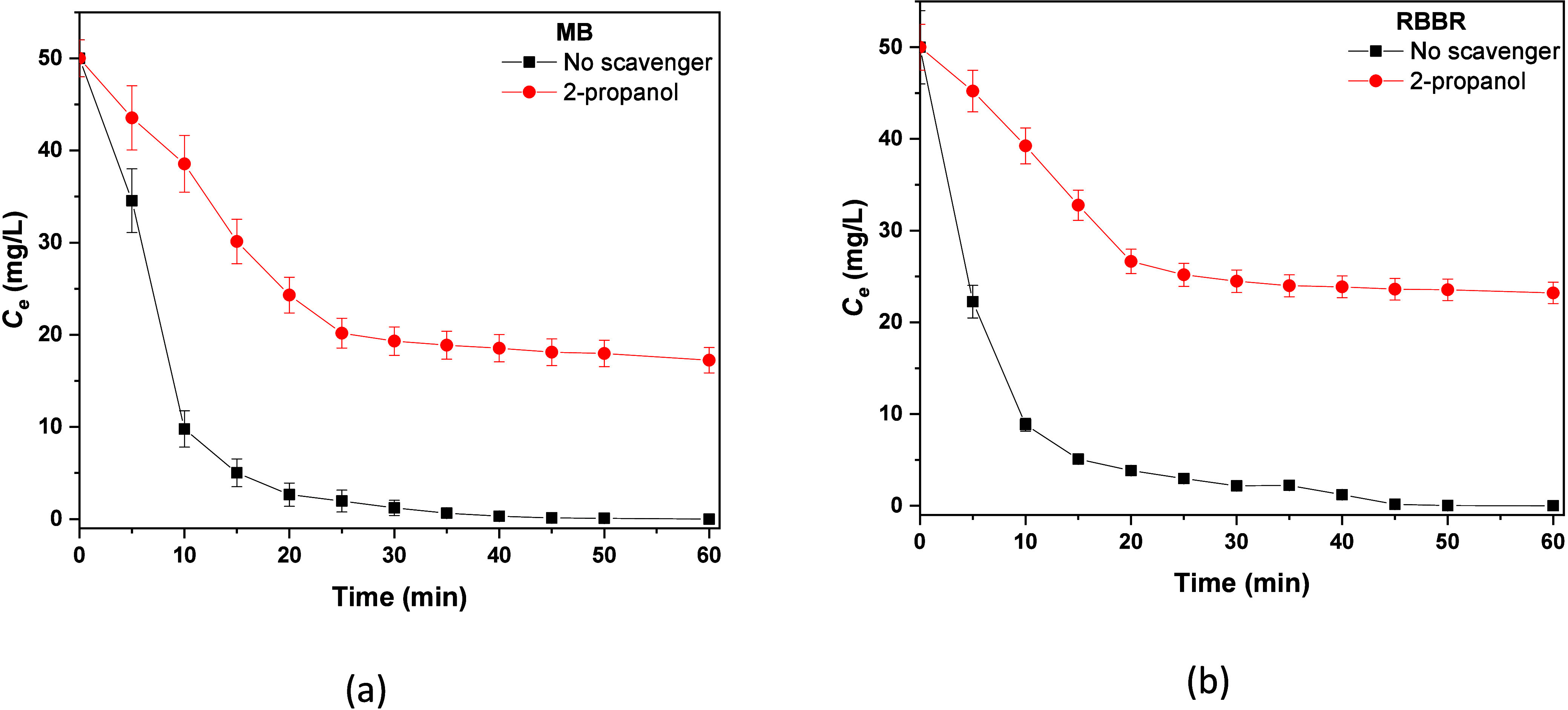
Radical quenching experiments for the degradation of (a) MB and (b) RBBR.

### 3.8 Real Wastewater

Another important and practical factor that was evaluated is the application of O_3_@NBs in real textile wastewaters. The properties of the textile effluent were reported in a previous study of our group.^4^ The results of dye removal (%) are presented in Figure 12a, along with total dissolved solids (TDSs) removal in Figure 12b and the obtained UV–vis spectra after each measurement. The λ_max_ used for the calculation of the dye removal is 605 nm, which presented the highest peak value. As can be seen (Figure 12a), the results seem very positive, since there was a fast removal of 35% in the first 5 min, followed by a gradual increase in the efficiency, achieving ∼75% removal after 60 min. The results from TDS removal were also exceptional, managing to remove >70% of the solids present in the solution. These results indicate that the resulting O_3_@NBs obtained by hydrodynamic cavitation can be successfully employed in real wastewater scenarios to produce clean and safe waters. Similar results have been presented in similar studies where NBs were employed in real wastewater samples.^77,78^

**Figure 12:**
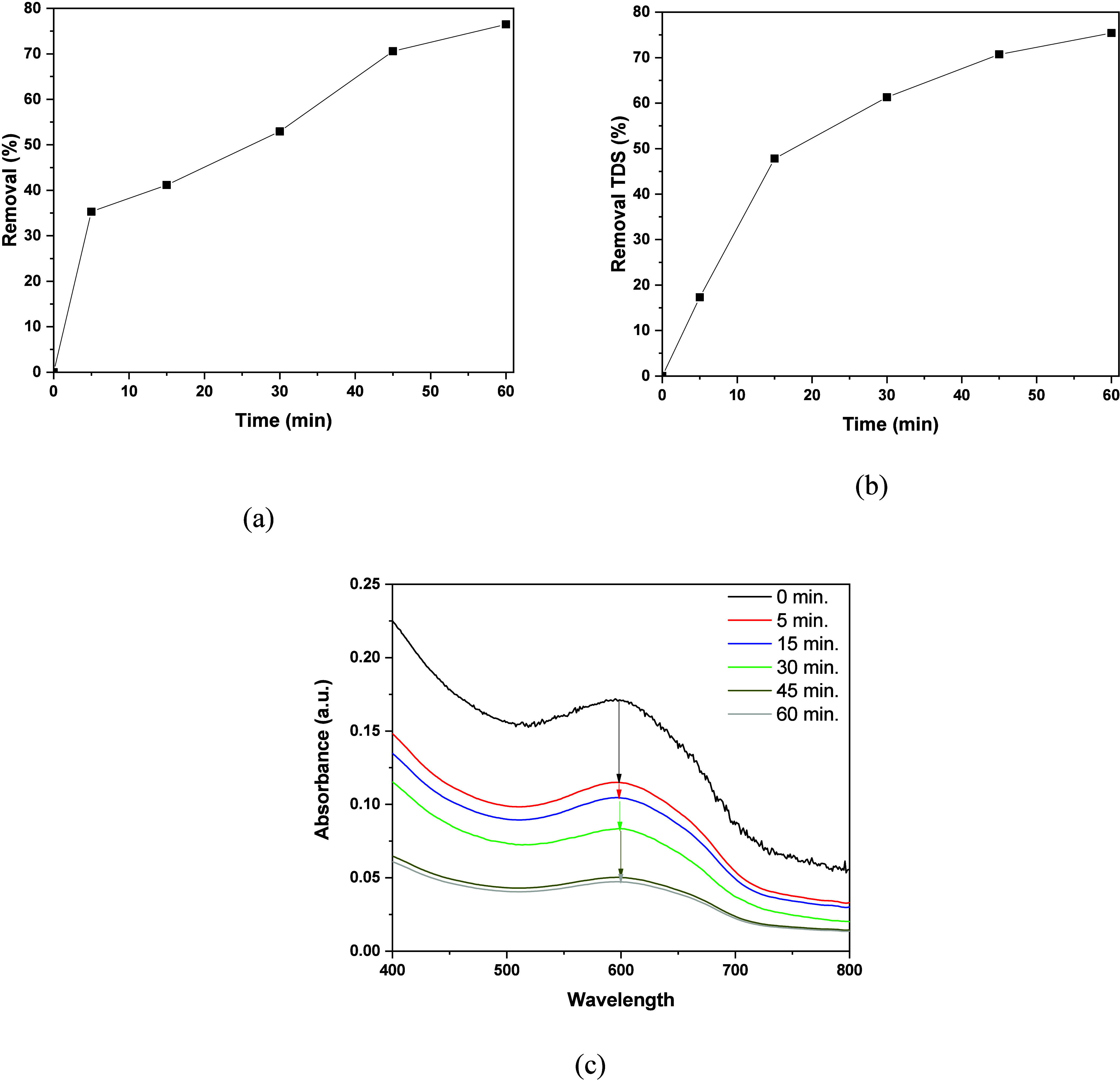
Removal of (a) dyes from real textile wastewater, (b) TDS removal, and (c) UV–vis spectra of wastewater after each time interval.

### 3.9 Comparative Studies

Comparative studies of NBs and MNBs have unveiled novel insights into their unique behaviors and synergistic applications, emphasizing their transformative potential across diverse fields. Li et al. generated air NBs through HC for the removal of hydroquinone. They managed to achieve more than 90% removal, at 7.38 pH. The equilibrium for this removal was met within 2 h, with initial concentration being 50 mg/L.^32^ In a similar study, Air MNBs were synthesized by using nanopore diffusion. They were extremely efficient, since they managed to degrade 100% of tetracycline at pH 4 at 15 min.^58^ Xiang et al. obtained Air MNBs, using MNB generator, to eliminate phenols from wastewaters.^79^ At 540 min, equilibrium was met, where 100% of initial concentration was removed. In another study by Koundle et al., they used a ceramic membrane, to produce O_3_ NBs.^54^ Degradation experiments were performed, leading to 10 min as the optimal degradation time for 100% removal, at a starting concentration of 10 mg/L. Most of the aforementioned works highlighted that the main degradation mechanism includes ROS production in water. In our study, three different types of gases were examined under various conditions (flow rate, pH). NBs were generated through HC with the purpose of eliminating MB and RBBR. In both cases O_3_@NBs managed to remove 100% under all conditions with rapid degradation kinetics. These results are summarized in Table 1.

**Table 1:** Comparative Studies Employing NBs/MNBs

Bubbles	Bubble generation	Pollutant	Removal (%)	Degradation params	Equilibrium time (min)	Ref
Air NBs	HC	Hydroquinone	91.25	pH 7.38; *C*_0_, 50 mg/L	120	^32^
Air MNBs	MNB generator	Phenol	100	*C*_0_, 400 mg/L	540	^79^
O_3_ NBs	Nanopore diffusion	As(III)	98	pH 3; *C*_0_, 1 mg/L	20	^70^
O_3_ NBs	Ceramic membrane	MB	100	pH 7.1; *C*_0_, 10 mg/L	10	^54^
O_3_ NBs	Nanopore diffusion	Tetracycline	100	pH 4; *C*_0_, 100 mg/L	15	^58^
O_3_@NBs	HC	MB		Flow rate, 0.3 L/min; pH 12; *C*_0_, 10 mg/L	20	This study
		RBBR		Flow rate, 0.3 L/min; pH 4; *C*_0_, 10 mg/L	15	This study

## 4 Conclusions

Hydrodynamic cavitation was employed to generate O_3_-, O_2_-, and Air@NBs for the degradation of a cationic (MB) and anionic (RRBR) dye. The gas flow rate played a significant role in both NB size and concentration, therefore affecting the degradation of organic pollutants. For the O_3_@NBs, 0.3 L/min managed to fully eliminate both studied pollutants at any pH level within 20 min. Effects of the degradation time for Air@NBs and O_2_@NBs revealed that they reach their plateau in 50 min for both dyes. Kinetics analysis showed that all three types of NBs fit well the first order model. The effect of the pH study showed that the aforementioned NBs were pH-sensitive, performing better in pH 12 and pH 4 for MB and RBBR, respectively. For the O_2_@NBs, the smaller bubbles demonstrated better degradation effectiveness, which may be attributed to their larger interfacial area per volume of gas and superior surface characteristics. The different degradation performance of MB and RBBR in processes with NBs can be understood through their molecular structures. Differences in such structures have important implications for adsorption performance, solubility and reactivity with ROS. Overall, NBs have shown a lot of promise in many areas, including wastewater treatment, the food industry, and agriculture, because of their unique properties. Looking ahead, research points to promising new applications, specifically in targeted drug delivery, catalysis, and advanced technologies for cleaning up the environment.

## Data Availability

Data will be made available on request.
